# The effect of social behavior change communication package on maternal knowledge in obstetric danger signs among mothers in East Mamprusi District of Ghana

**DOI:** 10.1186/s12992-017-0243-7

**Published:** 2017-03-21

**Authors:** Mahama Saaka, Paul Aryee, Robert kuganab-lem, Mohammed Ali, Abdul Razak Masahudu

**Affiliations:** 1 School of Allied Health Sciences, University for Development Studies, P O Box TL 1883, Tamale, Ghana; 2 2Catholic Relief Services (Ghana) Program, Tamale, Ghana

**Keywords:** Behavior change communication, Obstetric danger signs, Knowledge of danger signs, Neonatal danger signs, Child birth, Postpartum, East Mamprusi District, Ghana

## Abstract

**Background:**

An understanding of maternal knowledge of the danger signs of obstetric and newborn complications is fundamental to attaining universal health coverage. In Northern Ghana, where maternal and newborn morbidity and mortality is high, little is known about the current knowledge level and associated determinants of these danger signs. This study assessed the effect of social behavior change communication (SBCC) package on knowledge of obstetric and newborn danger signs among mothers with children under 24 months of age.

**Methods:**

This study used a non-randomized controlled community-based intervention design with pre and post-intervention household surveys in the intervention and comparison communities of the East Mamprusi District in Ghana. The study population were selected using a two-stage cluster sampling procedure.

**Result:**

Only 521 (51.1%), 300 (29.4%) and 353 (34.6%) of the study participants knew at least three key danger signs during pregnancy, delivery and postpartum period respectively.

The intervention had a positive effect on maternal knowledge of danger signs. Compared to their counterparts in the comparison communities, women in the intervention communities were about 2.6 times (AOR  =  2. 58 [CI: 1.87, 3.57]), 3.4 times (AOR  =  3.39 [CI: 2.31, 4.96]) and 2.2 times (AOR  =  2.19 [CI: 1.68, 2.84]) more likely to have higher knowledge of danger signs of childbirth, postpartum and neonate, respectively.

Having sought postnatal services at least once was significantly associated with the mentioning of at least three danger signs of postpartum (AOR  =  3.90 [CI: 2.01, 7.58]) and childbirth (AOR  =  1.75 [CI: 1.06, 2.85]).

**Conclusion:**

There was a significant contribution of social and behavioral change communication as an intervention to maternal knowledge in obstetric danger signs after adjusting for confounding factors such as antenatal and post-natal care attendance. Therefore, provision of information, education and communication targeting women on danger signs of pregnancy and childbirth and associated factors would be an important step towards attaining universal health coverage.

## Background

On daily basis, an approximate number of 830 women die from preventable causes related to pregnancy and childbirth and almost all of these deaths occur in developing countries [1].

This notwithstanding, there is evidence that shows that low utilization of services, such as supervised deliveries and post-natal care continue to persist even where financial and geographic access is adequate [2], thereby making progress towards attainment of significant improvement in maternal and newborn health a big challenge to most developing countries including Ghana. Developing countries account for approximately 99% of the global maternal deaths in 2015, with sub-Saharan Africa alone accounting for roughly 66% [3].

Apart from poor quality of health services, socio-cultural factors in the form of practices, rituals, attitudes and beliefs (PRABs) have been identified as key contributors to the poor health seeking behaviors and has engaged the attention of Ghana’s Ministry of Health (MoH) over the past years [4–6]. In particular, a high maternal and neonatal death rates in East Mamprusi District of Northern Ghana were reported to be associated with household PRABs as well as non-recognition of danger signs and lack of timely decisions to access services, which all increase risks of obstetric complications [7]. Evidence suggests, however, that social norms and practices can be changed through the communication of culturally contextualized messages designed to support families in modifying high risk practices [8].

Adequate maternal knowledge of the danger signs associated with pregnancy, labour and the neonate is fundamental to utilization of maternal and newborn care (MNC) services [9–13]. Poor knowledge of newborn danger signs delays care seeking and ultimately greater risk of death. Evidence suggests that raising awareness for pregnant women to recognize these obstetric danger signs would improve early detection of problems and reduces the delay in deciding to seek obstetric care during labor, delivery and early postpartum period [14–17].

To address unhealthful social norms, attitudes and practices, lack of knowledge and poor demand for preventive health services among key population groups in the East Mamprusi District, a comprehensive community-based social and behavior change communication (SBCC) intervention trial was implemented by the Catholic Relief Services (CRS) and its collaborators to improve uptake of maternal and newborn health care services. This paper reports on the effect of the intervention on knowledge in obstetric danger signs among mothers who had given birth in the past 24 months prior to the study.

## Methods

### Survey design, population and sampling

The paper is based on data that were obtained as part of an intervention to evaluate the effectiveness of social and behaviour change communication (SBCC) through empowered community leaders to improve uptake of essential maternal and newborn care (MNC) services in the East Mamprusi district of Northern Ghana.

A “pretest-posttest non-equivalent groups design” was used. Two cross-sectional household surveys at baseline and end point were carried out in both the intervention and comparison areas district to measure change in key indicators. The baseline survey was carried out in February 2012 and the end line survey was conducted in July 2015.

The main outcome of this study was to increase the proportion of institutional deliveries. This outcome indicator was used to calculate a sample size of 1020 (510 per study arm). The sample was set to detect a 15% difference in the comparison (43%) and intervention areas (58%). Given the sampling method, a design effect of 2.0 was selected. Power (1-β) and statistical significance (α) were set at 90% and 0.05 respectively. A non-response of 10% was also considered in the sample size calculation.

### Study Population and Sampling

The primary respondents comprised women of reproductive age who delivered within the last 2 years. Two sub districts which were geographically far apart but of similar socio-economic, demographic and health care characteristics were selected as intervention and comparison areas. A two-stage cluster sampling method was applied in each sub district to select the communities using probability proportional to size (PPS). Thirty clusters (i.e. communities) were selected randomly in the first stage from each study arm. In the second stage, eligible index households were selected in each cluster using systematic random sampling. Households with at least one woman who had delivered in the past 24 months were eligible for selection.

In each cluster, a complete list of all households was compiled serially and systematic random sampling used in selecting study participants. In the first step, the total number of households in a cluster was divided by the expected sample size of 17 to give the sampling interval. The first household was randomly selected by picking any number from 1 to the calculated sampling interval. Subsequent selections were made by adding the sampling interval to the selected number in order to locate the next household to visit. If the selected household did not have a target respondent, then the next household was selected using the systematic sampling procedure. This was done until the sample size was obtained.

### Description of interventions

A community-based non-randomized cluster intervention was implemented involving two arms; one intervention study area and one comparison study area. The Comparison Group received only standard maternal health services. The Intervention Group received an innovative intervention in addition to the standard maternal health services.

The innovative intervention in this study was based on evidence which suggests, that social norms and practices can be changed through the social and behaviour change communication (SBCC) which focuses on the community as the unit of change. On the basis of this, key community leaders (KCLs) including chiefs, queen mothers, religious leaders and traditional healers were mobilized to constitute what we termed ‘Council of Champions’ (CoCs) who assisted in delivering messages designed to support families in modifying high risk practices and delivered through interpersonal interactions involving reasoning and negotiation between frontline healthcare workers and target populations at the household and community level. The CoCs comprised 5-7 most influential community members who were trained and regularly supervised by the project.

These KCLs are also the custodians of the norms and values of their people and exert significant influence on various household decision makers whose actions influence the health of women and children within their communities [18, 19].

The intervention whose details have been published [20] was implemented from 2012 through 2015. The main project intervention package was a social and behavior change communication (SBCC) strategy which sought to bring about significant and sustainable improvements in maternal and newborn outcomes.

Delivery of health and nutrition messages through behaviour change communication (BCC), training, outreach and counselling were the key activities undertaken by the project. In the intervention communities, community leaders and volunteers were trained to identify and offer preventive and promotive care and counseling to pregnant and postpartum women. Health facility strengthening was done in all facilities to improve quality of care. Community health workers (CHWs) were trained and supported to promote early and regular ANC attendance.

### Variables and measurement

This study measured maternal knowledge in obstetric danger signs. Mothers were asked about risks associated with frequent pregnancies, signs and symptoms during pregnancy, delivery, newborn and postpartum danger signs which demanded seeking immediate care from the health facility or health workers.

A categorical variable was created to indicate the mother’s knowledge of such danger signs. A score of 1 was assigned if a mother could mention any of a listed number of danger signs. Knowledge of women about danger signs were measured by the total number of correct spontaneous answers to 8 items on knowledge of pregnancy danger signs, 7 items on knowledge of childbirth danger signs, 9 items on knowledge of postpartum danger signs and 10 items on newborn danger signs. Multiple responses were possible and spontaneous knowledge in this study refers to the respondent’s mentioning a sign without being given clues. The mothers were categorized into two based on the total scores obtained: Mothers who could mention 0–2 and those who could mention three or more danger signs. The category ‘knows up to 2 danger signs’ was used as the reference category.

### Assessment of Adequacy of Antenatal Care Service Utilization

The overall adequacy of prenatal care was measured using the Adequacy of Prenatal Care Utilization Index (APNCU) [21], modified according to the recommendation by the World Health Organization (WHO) for developing countries. The WHO recommends that for normal pregnancies, antenatal care (ANC) should consist of at least four visits during the course of the pregnancy, the first of which should occur within the first trimester. This measure involves both timing and number of prenatal care visits. Consequently, ANC was considered adequate if the women started ANC within the first trimester and made at least four visits. A woman who initiated ANC later than the first trimester but made at least four visits was thus classified as having inadequate ANC.

### Assessment of essential newborn care practices

In determining good essential newborn practices, composite indices were created: (i) Safe cord care (defined as use of a clean cutting instrument to cut the umbilical cord plus clean thread to tie the cord plus no substance applied to the cord); (ii) Optimal thermal care (defined as baby wrapped within 10 min of birth plus baby being dried/wiped immediately after birth); and (iii) Good neonatal feeding practices (defined as initiating breastfeeding within the first 1 h after birth, plus no prelacteal given and colostrum fed to the child). These composite variables were then dichotomized to Yes (all practices present) or No (one or more practices missing).

### Assessment of negative maternal and newborn care health (MNCH) rituals and beliefs

Potential negative maternal and newborn care health (MNCH) rituals and beliefs were assessed using the Likert-type of questions which elicited responses from respondents on their position regarding different rituals and birthing practices. Respondents were specifically asked how much they agree or disagree with the following statements:

i. A woman who is pregnant for the first time should only seek ANC services after she has announced her pregnancy to the community (Prisibu).
ii. Charcoal, oil, cow dung, a concoction or cosmetic pomades should be applied to help heal the cord of newborns.
iii. Before a woman in labor can go to the facility to deliver, the gods or ancestors should be consulted.
iv. A woman who delivers her baby at home is more of a “real woman” than a woman who delivers in a health facility.
v. After the birth of a baby, the mother should not go to the health facility until the naming ceremony has been performed.

### Data Collection

The data were collected using predesigned and pretested semi-structured questionnaire. Targeted eligible women were interviewed in the local language in their homes. Data collected included socio-economic conditions of household, nutritional status, maternal and newborn care practices, and health services utilization.

In order to ensure reliability and validity of data collected, all field assistants with a minimum qualification of Senior High School were given training for 3 days. The content of the training included objectives and methodology, standard measurement procedures, data recording, recruitment, administration of questionnaires and supervision.

### Data Analysis

The analysis of data took into account the complex design of multi-stage cluster surveys. All quantitative data were coded for statistical analysis using SPSS Complex Samples module for windows 18.0 (SPSS Inc, Chicago). This was done in order to make statistically valid population inferences and computed standard errors from sample data. Design weights were added to each region’s sample data (that is, total population divided by number of respondents) to perform weighted analysis.

The unit of analysis employed in this study is reproductive women with a child less than 24 months who were asked information on danger signs. A woman was considered knowledgeable when she could mention at least three recognizable danger signs for each of the critical periods of pregnancy, delivery, neonatal and postpartum.

The impact of the intervention was assessed using difference-in-difference (DID) analysis and measures of association (odds ratio). DID analyses allow for the determination of whether the intervention households did better than comparison households while taking into account any initial differences between the groups at baseline. By doing so it controls for any changes that took place in the project area that are not related to project interventions or that are only indirectly related to them through spillover effects.

Bivariate and multiple logistic regression analyses were performed to explore the knowledge levels and factors determining maternal knowledge on obstetric and newborn danger signs. The association between dependent variables and independent variables was determined using multiple logistic regressions modeling, which included all potential socioeconomic, and demographic confounders that were significant at *p* values < 0.05 in the bivariate analysis. The associations between knowledge on key danger signs of obstetric complication during the four periods (pregnancy, childbirth, neonatal and postpartum) and each independent variable were presented as adjusted odds ratios (AOR) with 95% confidence intervals (CI). A CI was considered statistically significant when the interval between the upper and lower values did not include one.

### Ethics approval and consent to participate

Approval for the conduct of this study was given by the Institutional Review Board (IRB) of the School of Medicine and Health Sciences, University for Development Studies (Reference no. SMHSER0001). Study participants were free to refuse or withdraw from the study at any time without any penalty. The study’s purpose and objectives were explained to each participant prior to interview. Informed consent was sought from all study participants before the commencement of any interviews or study activity. Additionally, participants who were literates signed the consent form but those who could not read made a thump-print after obtaining verbal explanation from the interviewer.

No biological sample was obtained as a part of the data collection. Data were kept strictly confidential and no personal identifiers were put on the questionnaires.

## Results

### Comparison of socio-demographic characteristics at baseline

At base-line, a total of 1003 respondents were interviewed; 510 from the Intervention Area (that is, Sakogu Sub-district) and 493 from the Comparison Area (Langbinsi Sub-district). There were significant differences between the two areas with respect to the age distribution of the children and ethnicity of the mothers. A greater proportion of women in the intervention than in the comparison communities were at least 6 km away from a health facility.

Both intervention and nonintervention groups were comparable in terms of age of mothers, educational level and parity. There were also significant differences in maternal knowledge in obstetric danger signs at baseline (Table 1).

**Table 1:** Comparison of socio-demographic characteristics of mothers having children less than 24 months in comparison versus intervention communities at baseline (*N* = 1003)

Characteristic	Intervention (*n* = 510)	Comparison (*n* = 493)	*p*-value
	n (%)	n (%)	
Age of mother (years)			
17–24	156 (30.6)	138 (28.0)	0.07
25–35	267 (52.4)	291 (59.0)	
35^+^	87 (17.0)	64 (13.0)	
Age of child (months)			
0–5	104 (20.4)	130 (26.4)	0.02
6–11	124 (24.3)	133 (27.0)	
12–23	282 (55.3)	230 (46.7)	
Educational level			
None	411 (80.6)	386 (78.3)	0.63
Basic (Primary or JHS)	91 (17.8)	97 (19.7)	
Senior high school or higher	8 (1.6)	10 (2.0)	
Ethnicity			
Mampruli	244 (47.8)	276 (56.)	<0.001
Moar	96 (18.8)	51 (10.3)	
Kusal	30 (5.9)	1 (0.2)	
Likpakpa	89 (17.5)	2 (0.4)	
Other	51 (10.0)	163 (33.1)	
Parity			
Primiparous	84 (16.5)	92 (18.7)	0.06
Secundipara	92 (18.0)	113 (22.9)	
Multiparous	334 (65.5)	288 (58.4)	
Distance of Health Facility to Home (Km)			
0–5	303 (45.7)	360 (54.3)	<0.001
6–10	106 (65.8)	55 (34.2)	
At least 7	95 (62.1)	58 (37.9)	
Maternal knowledge in least 3 danger signs			
Danger signs during pregnancy	78 (41.9)	108 (58.1)	0.007
Danger signs during delivery	194 (55.7)	154 (44.3)	0.024
Postpartum danger signs	257 (59.8)	173 (40.2)	<0.001
newborn danger signs	212 (75.2	70 (24.8)	<0.001

### Maternal Knowledge on danger signs and symptoms during pregnancy and delivery

Vaginal bleeding, severe abdominal pain and decreased foetal movement were frequently mentioned as the danger signs of pregnancy. Only 51.1% of respondents could mention at least three of the danger signs and symptoms during pregnancy that would prompt the mother to seek immediate care.

Similarly, the most commonly mentioned danger sign of childbirth was excessive vaginal bleeding by 485 (47.5%). Fast/difficult breathing and prolonged labour as danger signs were mentioned by a relatively smaller percentage of the respondents. The proportion of women who were able to mention three or more danger signs during delivery in the whole sample was 29.4% (Table 2).

**Table 2:** Maternal Knowledge on danger signs and symptoms during pregnancy and child birth (Multiple Responses)

Danger Sign	Frequency (*n*)	Percentage (*n*)
Danger signs during pregnancy		
Vaginal bleeding	486	47.6
Fast/difficult breathing of child	224	22.0
Fever	422	41.4
Severe abdominal pain	505	49.5
Headache/blurred vision	299	29.3
Convulsion	88	8.6
Foul smelling discharge/fluid from vagina	170	16.7
Baby stops moving	486	47.6
Knowledge of at least three of the above signs	521	51.1
Danger signs during child birth		
Convulsion	248	24.3
High fever	314	30.8
Heavy bleeding	485	47.5
Fast/Difficult Breathing	169	16.6
Retained Placenta	221	21.7
Headache/blurred vision	336	32.9
Prolonged Labour	188	18.4
Knowledge of at least three of the above signs	300	29.4

### Knowledge on danger signs during postpartum and neonatal periods

When the participants were asked to mention danger signs during postpartum, the most common spontaneously mentioned danger signs were severe abdominal pain and vaginal bleeding. The knowledge levels on the rest of the danger signs among the respondents were very low (Table 3).

**Table 3:** Maternal Knowledge on danger signs and symptoms during postpartum and newborn (Multiple Responses)

Danger Sign	Frequency (*n*)	Percentage (*n*)
Danger signs during postpartum		
Excessive vaginal bleeding	494	48.4
Fast/Difficult Breathing	216	21.2
High fever	361	35.4
Severe abdominal pain	595	58.3
Severe headache/blurred vision	283	27.7
Convulsions/loss of consciousness	173	17.0
Foul-smelling discharge from the vagina	103	10.1
Pain in calf	30	2.9
Verbalization/behavior that indicates she may hurt herself or the baby	13	1.3
Knowledge of at least three of the above signs	353	34.6
Newborn Danger signs		
Convulsion	384	37.6
Fever	563	55.2
Fast/Difficult Breathing	188	18.4
Baby feels cold	133	13.0
Poor suckling or feeding	416	40.8
Yellow palms/soles/eyes	94	9.2
Baby too small/too early	75	7.4
Swollen abdomen	118	11.6
Pus or redness of the umbilical stump, Eyes or skin	0	0.0
Unconsciousness	0	0.0
Knowledge of at least three of the above signs	383	37.5

Fever and poor suckling or feeding were the new born danger signs that were frequently mentioned and will prompt mothers to take their newborns to a health facility. The knowledge levels on other danger signs among the respondents were very low. Out of the ten newborn danger signs, only 37.5% of respondents could mention at least three danger signs.

### The effect of Social Behaviour Change Communication (SBCC) on maternal knowledge in obstetric danger signs

The difference-in-difference (DID) analysis comparing the changes over time for eligible intervention households and the comparison households indicates a significant improvement in respect of maternal knowledge in least 3 danger signs (Table 4).

**Table 4:** Difference-in-difference analysis: Percentage changes in maternal knowledge in least 3 danger signs from baseline to end-line

	Baseline			End-line			
Danger signs	Intervention	Comparison	Difference	Intervention	Comparison	Difference	DID
	*n* (%)	*n* (%)	(%)	*n* (%)	*n* (%)	(%)	(%)
Danger signs during pregnancy	78 (41.9)	108 (58.1)	−16.2^**^	306 (58.7)	215 (41.3)	17.4^***^	33.6^****^ CI:17.1, 50.2)
Danger signs during delivery	194 (55.7)	154 (44.3)	11.4^*^	212 (70.7)	88 (29.3)	41.4^***^	30.0^****^ CI:23.2, 36.6)
Postpartum danger signs	257 (59.8)	173 (40.2)	19.6^***^	272 (77.1)	81 (22.9)	54.2^***^	34.6^****^ CI:27.9, 41.0)
Newborn danger signs	212 (75.2)	70 (24.8)	50.4^***^	240 (62.7)	143 (37.3)	25.4^***^	−25.0^****^ CI:17.4, 32.4)

*p < 0.05; **p < 0.01; ***p < 0.001; ****p < 0.0001

There was a significant relative improvement in the proportion of women who could identify at least three danger signs during pregnancy, delivery and postpartum that needed the urgent attention of a health professional. However, the intervention had a negative impact on maternal knowledge in newborn danger signs.

Using binary logistic regression, there was significant improvement in maternal knowledge in least 3 danger signs (*P* < *0.001*) in the Intervention Group as compared to Comparison Group. Though there were significant differences in maternal knowledge in obstetric danger signs at baseline, these were not a significant determinants of maternal knowledge at follow-up (Table 5).

**Table 5:** Maternal knowledge in at least 3 obstetric danger signs in intervention group at follow-up survey (adjusted for baseline results)

Outcome	Crude Odds Ratio	95% CI	*P* value
Danger signs during pregnancy			
Comparison Group	Reference	Reference	Reference
Intervention Group	2.11	1.64–2.71	<0.001
Baseline knowledge	1.16	0.84–1.61	0.36
Danger signs during delivery			
Comparison Group	Reference	Reference	Reference
Intervention Group	3.34	2.49–4.47	<0.001
Baseline knowledge	0.94	0.70–1.26	0.67
Danger signs of during postpartum			
Comparison Group	Reference	Reference	Reference
Intervention Group	5.70	4.23–7.67	<0.001
Baseline knowledge	1.31	0.99–1.74	0.06
Newborn danger signs			
Comparison Group	Reference	Reference	Reference
Intervention Group	2.33	1.77–3.07	<0.001
Baseline knowledge	0.97	0.72–1.31	0.853

### Factors associated with knowledge of women on danger signs of pregnancy and childbirth

In a multivariate logistic regression analysis, the intervention, maternal age, uptake of postnatal services, and negative maternal and newborn care health (MNCH) rituals and beliefs were independently associated with the mentioning of at least three obstetric danger signs (Table 6).

**Table 6:** Association between knowledge of at least 3 danger signs during pregnancy childbirth, neonatal and postpartum periods and selected socio-demographic and obstetric characteristics

Variable	AOR (95% CI) danger signs of pregnancy	AOR (95% CI) danger signs during birth	AOR (95% CI) postpartum danger signs	AOR (95% CI) newborn danger signs
Treatment Arm				
Comparison	Reference	Reference	Reference	Reference
Intervention	1.90 (1.47, 2.45)^***^	2.58 (1.87, 3.57)^***^	3.39 (2.31, 4.96)^***^	2.19 (1.68, 2.84)^***^
Maternal Age (years)				
Under 25	Reference	Reference	Reference	Reference
25–34	0.91 (0.67, 1.22)	Not significant in final model	Not significant in final model	Not significant
≥ 35	1.52 (1.03, 2.23)^*^	Not significant in final model	Not significant in final model	Not significant
Adequacy of ANC				
No	Reference	Reference	Reference	Reference
Yes	Not significant	Not significant in final model	1.50 (1.04, 2.16)^*^	1.58 (1.19, 2.10)^*^
Having at least one wrong MNCH belief				
Yes	Reference	Reference	Reference	Reference
No	1.81 (1.40, 2.35)^***^	1.71 (1.26, 2.32)^**^	1.39 (1.02, 1.89)^*^	0.70 (0.53, 0.92)^*^
Uptake of postnatal services at least once in the first week after delivery				
No	Reference	Reference	Reference	Reference
Yes	Not significant in final model	1.75 (1.06, 2.85)^*^	3.90 (2.01, 7.58)^***^	1.54 (1.02, 2.32)^*^

^*^significant at *p* <0.05; ^**^significant at *p* <0.01; ^***^significant at *p* <0.001

AOR (95% CI): Adjusted odds ratio at 95% confidence level

The intervention had a positive effect on maternal knowledge of danger signs. Compared to their counterparts in the comparison communities, women in the intervention communities were about 2.6 times (AOR  =  2. 58 [CI: 1.87, 3.57]), 3.4 times (AOR  =  3.39 [CI: 2.31, 4.96]) and 2.2 times (AOR  =  2.19 [CI: 1.68, 2.84]) more likely to have higher knowledge of danger signs of childbirth, postpartum and neonate, respectively.

The other strong predictor of knowledge about the danger signs of postpartum and childbirth was patronage of postnatal services in the first week after delivery. Having sought postnatal services at least once was significantly associated with the mentioning of at least three danger signs of child birth (AOR  =  1.75 [CI: 1.06, 2.85]), postpartum (AOR  =  3.90 [CI: 2.01, 7.58]) and newborn danger signs (AOR  =  1.54 [CI: 1.02, 2.32]). Women who sought postnatal services in the first week after delivery were 3.9 times more likely to be knowledgeable in the dangers during the postpartum period, compared to women who did not.

Maternal age at interview showed an independent association only with knowledge of women in the danger signs of pregnancy. Mothers aged at least 35 years were 1.5 times (AOR  =  1. 52 [CI: 1.03, 2.23]) more likely to be knowledgeable about the danger signs of pregnancy as compared to those who were less than 25 years.

Women who received adequate ANC services were about 1.5 times (AOR  =  1.50 [CI: 1.05, 2.16]) and 1.6 times (AOR  =  1.58 [CI: 1.19, 2.10]) more likely to have higher knowledge of danger signs of postpartum and neonate as compared to their counterparts who did not get adequate ANC.

Women who reported no unhealthful social norms, beliefs, attitudes and practices were 1.8 times (AOR  =  1. 81 [CI: 1.40, 2.35]; 1.7 times (AOR  =  1. 72 [CI: 1.26, 2.32]; 1.4 times (AOR  =  1. 39 [CI: 1.02, 1.89]; and 30.0% (AOR  =  0. 70 [CI: 0.53, 0.92]) more likely to recognize danger signs during pregnancy, delivery, postpartum and newborn respectively.

Having at least one wrong maternal and newborn care health (MNCH) belief was negatively associated with the mother’s ability to recognize danger signs during pregnancy, delivery and postpartum. Some of the negative rituals and birthing practices were:

i. A woman who is pregnant for the first time should only seek ANC services after she has announced her pregnancy to the community (Locally known as “Prisibu”).
ii. Before a woman in labour can go to the facility to deliver, the gods or ancestors should be consulted.
iii. A woman who delivers her baby at home is more of a “real woman” than a woman who delivers in a health facility.
iv. After the birth of a baby, the mother should not go to the health facility until the naming ceremony has been performed.

Other independent variables including parity, marital status, occupation, place of delivery and religion were not associated with awareness of a danger sign during pregnancy, delivery and after delivery in the bivariate logistic regression analysis, and were therefore not included in the multivariate logistic regression analysis.

### Association between improved maternal knowledge in obstetric danger signs and uptake of maternal and newborn health services

As shown in Table 7, improved knowledge of obstetric danger signs was associated with uptake of ANC services and adoption of essential newborn care practices but not patronage of health facilities for delivery. Association between maternal knowledge in danger signs and maternal and newborn practices/behaviours Table 8 shows the association between maternal knowledge in danger signs and maternal and newborn practices/behaviours..

**Table 7:** Association between improved maternal knowledge in obstetric danger signs and uptake of maternal and newborn health services (Bivariate analysis)

Variable	Category		Place of birth		
Knowledge of at least three dangers signs		*N*	Home *n* (%)	Health Facility *n* (%)	Test statistic
During pregnancy	<3	493	64 (13.0)	429 (87.0)	Chi-square (*χ* ^*2*^) = 0.25, *p* = 0.61
	≥3	519	73 (14.1)	446 (85.9)	
During child birth	<3	712	81 (11.4)	631 (88.6)	(*χ* ^*2*^) =9.58, *p* = 0.002
	≥3	300	56 (18.7)	244 (81.3)	
During postpartum	<3	659	76 (11.5)	583 (88.5)	(*χ* ^*2*^) =6.49, *p* = 0.011
	≥3	353	61 (17.3)	292 (82.7)	
Newborn danger signs	<3	633	75 (11.8)	558 (88.2)	Chi-square (*χ* ^*2*^) = 4.1, *p* = 0.04
	≥3	379	62 (16.4)	317 (83.6)	
			Adequacy of ANC uptake?		
			No	Yes	
During pregnancy	<3	484	249 (51.4)	235 (48.6)	(*χ* ^*2*^) = 10.1, *p* = 0.001
	≥3	494	204 (41.3)	290 (58.7)	
During child birth	<3	688	365 (53.1)	323 (46.9)	(*χ* ^*2*^) = 42.3, *p* < 0.001
	≥3	290	88 (30.3)	202 (69.7)	
During postpartum	<3	633	364 (57.5)	269 (42.5)	(*χ* ^*2*^) = 90.28, *p* < 0.001
	≥3	345	89 (25.8)	256 (74.2)	
Newborn danger signs	<3	612	318 (52.0)	294 (48.0)	(*χ* ^*2*^) = 20.9, *p* < 0.001
	≥3	366	135 (36,9)	231 (63.1)

**Table 8:** Association between maternal knowledge in danger signs and maternal and newborn practices/behaviours

Variable	Category				
Knowledge of at least three dangers signs			Good neonatal feeding practices?		
			No	Yes	
During pregnancy	<3	499	240 (48.1)	259 (51.9)	(*χ* ^*2*^) =2.28, *p* = 0.13
	≥3	521	226 (43.4)	295 (56.6)	
During child birth	<3	720	359 (49.9)	361 (50.1)	(*χ* ^*2*^) = 17.2, *p* < 0.001
	≥3	300	107 (35.7)	193 (64.3)	
During postpartum	<3	667	366 (54.9)	301 (45.1)	(*χ* ^*2*^) = 65.54, *p* < 0.001
	≥3	353	100 (28.3)	253 (71.7)	
Newborn danger signs	<3	637	314 (49.3)	323 (50.7)	(*χ* ^*2*^) = 8.9, *p* = 0.003
	≥3	383	152 (39.7)	231 (60.3)	
			Safe cord care?		
			No	Yes	
During pregnancy	<3	493	388 (78.7)	105 (21.3)	(*χ* ^*2*^) = 9.04, *p* = 0.003
	≥3	507	357 (70.4)	150 (29.6)	
During child birth	<3	711	571 (80.3)	140 (19.7)	(*χ* ^*2*^) = 43.7, *p* < 0.001
	≥3	289	174 (60.2)	115 (39.8)	
During postpartum	<3	661	540 (81.7)	121 (18.3)	(*χ* ^*2*^) = 53.1, *p* < 0.001
	≥3	339	205 (60.5)	134 (39.5)	
Newborn danger signs	<3	631	501 (79.4)	130 (20.6)	(*χ* ^*2*^) = 21.59, *p* < 0.001
	≥3	369	244 (66.1)	125 (33.9)	

## Discussion

In Northern Ghana, where maternal and newborn morbidity and mortality is high, little is known about the current knowledge level on obstetric, newborn danger signs and associated determinants among women of reproductive age. This study has provided evidence that may be useful in planning and monitoring the impact of behavior change communication programme efforts by Maternal and Child Health programmes that seek to increase knowledge of danger signs among women and to prevent delays in seeking MNCH services/care.x

### Maternal Knowledge on risks and danger signs associated with pregnancy, delivery and newborn and postpartum

An understanding of maternal knowledge of the danger signs of obstetric and newborn complications is fundamental to attaining universal health coverage. In this study a woman was considered knowledgeable if she could mention at least three recognized danger signs for each of the critical periods of pregnancy, delivery, postpartum and neonate.

Consistent with other studies, a significant proportion of mothers were not knowledgeable about the danger signs of pregnancy, childbirth, postpartum and the newborn in the district.

Several studies have also revealed that mothers from some developing countries including Sri Lanka, Kenya, Uganda and Nepal had unsatisfactory levels of knowledge in the recognition of newborn danger signs [22–26]. A community based cross-sectional study conducted in Tanzania showed that about half of the study subjects knew at least one obstetric danger sign [27], and in Kenya, only 27.9% of women attending ANC were not informed about danger signs in pregnancy [16]. This finding is also consistent with studies conducted in Ethiopia in which 30.9% of respondents mentioned at least two danger signs of pregnancy [28, 29]. This indicates a significant number of pregnant women who do not have the knowledge are likely to delay in deciding to seek care.

Excessive vaginal bleeding was the most common and spontaneously mentioned danger sign of pregnancy, childbirth and during the postpartum period, whilst severe abdominal pain and decreased foetal movement were also frequently added as danger signs of pregnancy. Severe abdominal pain was also mentioned as a danger sign during postpartum, with fever and poor suckling or feeding more frequently mentioned as new born danger signs.

Higher awareness of vaginal bleeding as a danger sign has been reported in Karachi, Pakistan [30]. Some studies have indicated that vaginal bleeding after delivery is most commonly recognized as a danger sign perhaps because it is the most visible sign and the most common cause of maternal death immediately after delivery [11, 31].

Recognizing danger signs by pregnant women could improve early detection of problems and reduce the delay in deciding to seek obstetric care [14–16]. Thus, the importance of creating awareness on danger signs among pregnant women and family members to take appropriate steps to ensure a safe birth and to seek timely skilled care in emergencies cannot be over-emphasized.

The strong predictors of knowledge of women about danger signs of labor and childbirth were adequate ANC, postnatal services and having at least one wrong maternal and newborn care health (MNCH) belief. Maternal age was an independent predictor only with knowledge of women about the danger signs of pregnancy. Increased knowledge levels among older women may be related to their own experiences with pregnancy. This implies that young women in their first pregnancy may need more consideration when providing counseling and health education.

The evidence from this study suggests that exposure to adequate ANC services was critical and more likely to increase mothers’ awareness and knowledge regarding danger signs. This finding is in consonance with earlier studies which have shown that health education during antenatal care improves mothers’ knowledge about obstetric danger signs [32, 33].

However, the study revealed that only a small proportion of women really received adequate ANC services. The health and local community authorities would have to put measures in place to promote uptake of ANC services early in pregnancy. In addition to the antenatal and postnatal care counseling, other channels of spreading key messages including community-based radio broadcasts should be considered. Furthermore, it is important for educational messages to target women’s groups, husbands, mothers in-law, and other family members, who play an important role in the decision making process [34, 35].

The postnatal period begins immediately after the birth of the baby and extends up to 6 weeks (42 days) after birth [36]. The immediate postpartum period is a dangerous time for both the mother and newborn [36–38] and between 50 and 71% of maternal deaths happen during postpartum period, particularly in the first few hours [39]. Recognizing danger signs and symptoms during this period is therefore critical for the health of mother and newborn. In this study, patronizing postnatal services at least once during the first week after delivery was the most important predictive factor for increased awareness of danger signs in the postpartum period. This finding is corroborated in at least one study [40]. This can be explained by the fact that women would have become aware of obstetric danger signs through education received at postnatal care centres as this issue is often among the topics for discussion. In spite of this, only 31.4% of mothers in the study sample utilized postnatal care service at least twice in the first week of delivery.

Level of education was not an important determinant of the mother’s ability to mention at least three danger signs during pregnancy and childbirth in this study population and this may be due to the very large numbers of non-educated women in our sample. However, a number of studies have shown strong association of mother’s educational attainment with their knowledge of danger signs [41–44].

### Effect of behavior communication intervention on maternal knowledge of obstetric danger signs

The findings of the present study demonstrated that effective behavior communication intervention significantly improved in danger sign recognition.

The results of the study support findings of earlier studies in which BCC effectively improved obstetric danger sign recognition [45] though maternal knowledge of newborn care rather decreased in the intervention arm, compared to changes in the comparison arm in our sample.

Furthermore, such knowledge acquired positively associated with uptake of antenatal care services and adoption of essential newborn care practices. Improved knowledge of obstetric danger signs is expected to enhance utilization of maternal health services. This is because recognition of obstetric danger signs is a key factor in seeking preventive care or health promotion during pregnancy and childbirth.

Increased obstetric knowledge is reported to have enhanced greater use of facility delivery care [46]. Therefore, provision of information, education and communication targeting women on danger signs of pregnancy and childbirth would be an important step towards attaining universal health coverage.

Though increased obstetric knowledge was associated with adequate utilization of ANC services in the present study, it did not improve health facility delivery. This may be attributed to the absence of midwife in the intervention area. The midwife left the intervention area as a result of internal tribal conflict in the area during the project period.

This notwithstanding, some studies have shown that increased knowledge is not necessarily linked with behavior change [47].

## Conclusion

There was a significant contribution of social and behavioral change communication as an intervention to maternal knowledge in obstetric danger signs after adjusting for confounding factors such as antenatal and post-natal care attendance.

This study further showed that the proportion of mothers who could mention at least three danger signs associated with frequent pregnancies, delivery, postpartum and newborn was low. The implication of this low knowledge levels is that delays in deciding to seek emergency care would still persist. Therefore, measures including provision of information, education and communication should be put in place by the health and local community authorities to promote uptake of ANC services early in pregnancy. In addition to the antenatal and postnatal care counseling, other channels of spreading key messages including community-based radio broadcasts should target women’s groups, husbands, mothers in-law, and other family members, who play an important role in the decision making process. It is suggested that further studies on the quality of counseling on danger signs and utilization of health services be carried out in order to design appropriate training modalities for health workers.

### Limitations of the study

A number of factors may confound our findings. First of all, though there were significant differences in baseline characteristics between the study arms, these were accounted for using a difference-indifferences analysis. Secondly, responses were based on self-report and so measurement bias cannot also be ruled out. Finally, recall bias was more likely since women were asked for events which have already happened within the past 2 years prior to this study in spite of the fact that the youngest child and for that matter recent births were being referred to in the interviews.

## Abbreviations

ANC: Antenatal care
AOR: adjusted odds ratios
APNCU: Adequacy of Prenatal Care Utilization
MNC: Maternal and newborn care
MNCH: Maternal and newborn care health
PPS: Probability Proportionate to Population Size
PSU: Primary sampling unit
SBCC: Social and behaviour change communication
